# The BCR::ABL1 tyrosine kinase inhibitors ponatinib and nilotinib differentially affect endothelial angiogenesis and signalling

**DOI:** 10.1007/s11010-024-05070-5

**Published:** 2024-07-15

**Authors:** Darya Zibrova, Thomas Ernst, Andreas Hochhaus, Regine Heller

**Affiliations:** 1 Center for Molecular Biomedicine, Institute of Molecular Cell Biology, Jena University Hospital, Hans-Knöll-Straße 2, 07745 Jena, Germany; 2 Department of Hematology and Oncology, Jena University Hospital, Jena, Germany

**Keywords:** BCR::ABL1 inhibitors, Chronic myeloid leukaemia, Vascular side effects, Angiogenesis, Vascular endothelial cells

## Abstract

BCR::ABL1 inhibitors, the treatment of choice for the majority of patients with chronic myeloid leukaemia (CML), can cause vascular side effects that vary between agents. The exact underlying mechanisms are still poorly understood, but the vascular endothelium has been proposed as a site of origin. The present study investigates the effects of three BCR::ABL1 inhibitors, ponatinib, nilotinib and imatinib, on angiogenesis and signalling in human endothelial cells in response to vascular endothelial growth factor (VEGF). The experiments were performed in endothelial cells isolated from human umbilical veins. After exposure to imatinib, ponatinib and nilotinib, the angiogenic capacity of endothelial cells was assessed in spheroid assays. VEGF-induced signalling pathways were examined in Western blotting experiments using different specific antibodies. RNAi technology was used to downregulate proteins of interest. Intracellular cGMP levels were measured by ELISA. Imatinib had no effect on endothelial function. Ponatinib inhibited VEGF-induced sprouting, while nilotinib increased spontaneous and VEGF-stimulated angiogenesis. These effects did not involve wild-type ABL1 or ABL2, as siRNA-mediated knockdown of these kinases did not affect angiogenesis and VEGF signalling. Consistent with their effects on sprouting, ponatinib and nilotinib affected angiogenic pathways in opposite directions. While ponatinib inhibited VEGF-induced signalling and cGMP formation, nilotinib activated angiogenic signalling, in particular phosphorylation of extracellular signal-regulated kinase 1/2 (Erk1/2). The latter occurred in an epidermal growth factor receptor (EGFR)-dependent manner possibly via suppressing Fyn-related kinase (FRK), a negative regulator of EGFR signalling. Both, pharmacological inhibition of Erk1/2 or EGFR suppressed nilotinib-induced angiogenic sprouting. These results support the notion that the vascular endothelium is a site of action of BCR::ABL1 inhibitors from which side effects may arise, and that the different vascular toxicity profiles of BCR::ABL1 inhibitors may be due to their different actions at the molecular level. In addition, the as yet unknown pro-angiogenic effect of nilotinib should be considered in the treatment of patients with comorbidities associated with pathological angiogenesis, such as ocular disease, arthritis or obesity.

**Supplementary Information:**

The online version contains supplementary material available at 10.1007/s11010-024-05070-5.

## Background

Chronic myeloid leukaemia (CML), a myeloproliferative disorder, is caused by a chromosomal translocation that results in a fusion of two genes, the breakpoint cluster region protein (BCR) gene on chromosome 22 and the Abelson murine leukaemia viral oncogene homolog 1 (ABL1) gene on chromosome 9 [1]. The resulting *BCR::ABL1* gene encodes the oncoprotein BCR::ABL1, a constitutively active kinase that stimulates multiple pathways involved in cell survival and growth leading to the induction of malignant proliferation and inhibition of apoptosis. Consequently, the BCR::ABL1 kinase is a target for CML treatment [2]. Five ATP-competitive tyrosine kinase inhibitors (TKIs)—imatinib, dasatinib, nilotinib, bosutinib and ponatinib—and the allosteric TKI asciminib are approved for the treatment of CML in Europe. First-line treatment includes the first-generation TKI imatinib and the second-generation TKIs nilotinib, dasatinib and bosutinib. Imatinib treatment often leads to drug resistance caused by amino acid substitutions within the kinase domain of BCR::ABL1, which can be overcome by second- or third-generation TKIs. Nilotinib, dasatinib and bosutinib are active against most BCR::ABL1 mutants [3, 4], and ponatinib, a third-generation TKI, is active against all of them [5]. Accordingly, ponatinib and asciminib, which targets a completely different site, are approved for the treatment of patients who are resistant to prior TKI therapy, particularly those who carry a T315I mutation in the kinase domain of BCR::ABL1.

Imatinib has been shown to be the most selective BCR::ABL1 ATP competitive inhibitor, which is consistent with its clinical safety profile. Compared to imatinib, BCR::ABL1 inhibitors of second- and third-generations are characterised by more serious toxicity profiles (reviewed in [6]), with nilotinib and ponatinib causing mainly adverse vascular side effects [7]. For example, nilotinib and ponatinib treatments are associated with peripheral arterial occlusive disease and ponatinib causes systemic hypertension (reviewed in [7]).

The mechanisms underlying the cardiovascular toxicities caused by nilotinib or ponatinib are still poorly understood. Most likely, since these compounds bind to the ATP pocket within the kinase domain of BCR::ABL1, they may interact with similar domains in other kinases. Indeed, a number of studies using different methodologies have identified potential off-target kinases of BCR::ABL1 inhibitors [8–11]. According to these studies, each compound has a specific drug-protein interaction profile that may provide a mechanistic link to the different patterns of side effects. Kinases that may be strong candidates for off-target effects of BCR::ABL1 inhibitors, are discoidin domain receptor 1 (DDR1) kinase, SRC family kinases (e.g. FYN), TEC kinase, and the receptor tyrosine kinases ephrin receptor (EPH), platelet-derived growth factor receptors α and β (PDGFRα, β) and KIT [8–11] but their contribution to vascular side effects has not yet been validated. In addition, adverse effects of BCR::ABL1 inhibitors may also involve effects on wild-type ABL kinases. Previous studies have shown that deletion of endothelial ABL in mice increases endothelial cell apoptosis by interfering with angiopoietin/Tie2 protein kinase signalling and leads to increased vascular permeability via effects on the acto-myosin cytoskeleton [12, 13]. ABL kinase inhibition may therefore contribute to the vascular side effects of BCR::ABL1 inhibitors but, in general, little is known about the role of ABL in vascular cells.

Mechanistic studies investigating the vascular effects of BCR::ABL1 inhibitors are also limited. Ponatinib has been shown to induce apoptosis, reduce proliferation and migration, increase leukocyte adhesion, inhibit tube formation in human endothelial cells and impair endothelial progenitor cell function [14–16]. Nilotinib has been reported to upregulate pro-atherogenic adhesion proteins and leukocyte adhesion in human endothelial cells and to affect proliferation, migration, tube formation and viability [16, 17]. To further understand the vascular effects of BCR::ABL1 inhibitors and their underlying mechanisms, we profiled three BCR::ABL1 inhibitors, ponatinib, nilotinib and imatinib, in human endothelial cells with respect to their effects on signalling pathways and angiogenesis induced by vascular endothelial growth factor (VEGF), a potent endothelial survival factor and angiogenic stimulus. Our study shows that ponatinib and nilotinib alter angiogenesis in opposite directions while imatinib has no effect. Ponatinib inhibits VEGF-induced angiogenesis by blocking VEGF-triggered signalling whereas nilotinib induces spontaneous sprouting and potentiates VEGF-induced angiogenesis by activating angiogenic proteins, in particular Erk1/2. Our study provides further evidence that vascular endothelial cells are targeted by BCR::ABL1 inhibitors to induce individual off-target effects leading to functional changes in the vasculature.

## Methods

### Materials

Cell culture medium 199 (M199) was from Lonza (Verviers, Belgium). Recombinant human VEGF-165 was purchased from R&D Systems, Inc. (Minneapolis, MN, USA). Human plasma fibrinogen was acquired from Merck/Millipore (Darmstadt, Germany). Proteinase inhibitor mixture complete, EDTA-free, was purchased from Roche Diagnostics (Mannheim, Germany). Imatinib, nilotinib and ponatinib were from Cayman Chemical (Ann Arbor, MI, USA). All other reagents were from Sigma-Aldrich (Taufkirchen, Germany) unless otherwise stated.

### Antibodies

The following antibodies were from Cell Signalling Technology (Frankfurt, Germany): Total VEGFR2 (#2479), phospho(Y1175)-VEGFR2 (#2478), total VEGFR1 (#64,094), phospho(S1177)-eNOS (#9570), total Akt (#4691), phospho(S473)-Akt (#9271), total Erk (#9107), phospho(T202/Y204)-Erk (#9106), total AMP-activated protein kinase α (AMPKα) (#2532), phospho(T172)-AMPKα (#2531), total p38 (#9212), phospho(T180/Y182)-p38 (#4511), phospho(Y207)-Crk-L (#3181), phospho(Y1086)-EGFR (#2220), total EGFR (#2232). Total eNOS antibody (#610,297) and total ABL-1 antibody were purchased from BD Biosciences (Heidelberg, Germany). Total ABL-2 (#sc-81,154) and total Crk-L (#sc-365,092) antibodies were obtained from Santa Cruz Biotechnology (Dallas, TX, US). Total FRK antibody (#A95,485) was from Antibodies.com (Stockholm, Sweden). Horseradish peroxidase (HRP)-conjugated secondary antibodies to rabbit or mouse IgG were from Kirkegaard and Perry Laboratories, Inc. (Gaithersburg, MD, USA).

### Primary endothelial cells and treatment conditions

Human umbilical vein endothelial cells (HUVEC) were isolated from anonymously obtained umbilical cords in accordance with the Declaration of Helsinki “Ethical principles for Medical Research Involving Human Subjects” (1964). The study was approved by the ethics committee of the Jena University Hospital. The donors were informed and gave their written consent. Cells from 3 to 5 individual batches per experimental setting were used in the present study.

HUVEC were isolated and cultured on gelatine-coated culture flasks or dishes in M199 containing 17.5% fetal calf serum (FCS), 2.5% human serum, 100 U/ml penicillin, 100 µg/ml streptomycin, 680 µM glutamine, 25 µg/ml heparin, 7.5 µg/ml endothelial mitogen and 100 µM vitamin C (growth medium) as previously described [18]. Cells were seeded at a seeding density of 23.000/cm^2^ on 30 mm-dishes for signalling experiments and cGMP determination. For transfection with siRNA, 18.000 cells/cm^2^ were seeded on 90 mm-dishes. Experiments were performed with cells from the first or second passage.

Before treatment with TKIs, HUVEC were serum-starved for 4 h in M199 containing 0.25% human serum albumin (M199/HSA). Preincubation with TKI or vehicle (DMSO) was performed for 30 min in Hepes buffer (10 mM Hepes (pH 7.4), 145 mM NaCl, 5 mM KCl, 1 mM MgSO_4_, 1.5 mM CaCl_2_, 10 mM glucose) supplemented with 0.25% human serum albumin (Hepes/HSA). The cells were then stimulated with VEGF (50 ng/ml, 5, 10 min or 20 min as indicated) or left unstimulated.

### Determination of cGMP levels

HUVEC monolayers were preincubated with the indicated TKI for 30 min in Hepes/HSA containing 0.5 mM isobutylmethylxanthine (IBMX). They were then stimulated with VEGF (50 ng/ml) for 20 min in the same medium or left unstimulated. Cells were lysed in 0.1 M HCl and intracellular cGMP levels were determined using the competitive ELISA kit (581,021, Cayman Chemical, Ann Arbor, MI, USA) according to the manufacturer’s instructions.

### Small interfering RNA (siRNA)-mediated gene knockdown

The siRNA duplex oligonucleotides used in this study were based on the human cDNAs encoding ABL1, ABL2, DDR1 and VEGFR1. ABL1-, ABL2-, DDR1- and VEGFR1-specific SMARTpool siRNA reagents (L-003100-00-0005, L-003101-00-0005, L-003111-00-0005, M-003136-03-0005, respectively) and a non-specific SMARTpool control siRNA (D-001810-10-20) were purchased from Dharmacon/GE Healthcare (Chicago, IL, USA). HUVEC seeded on 90 mm-diameter dishes 24 h before transfection were 50% confluent when siRNA was added. The amount of siRNA duplexes applied was 4 μg/dish. Transfection was performed using the amphiphilic delivery system SAINT-RED (Synvolux Therapeutics B.V., Groningen, The Netherlands) according to the manufacturer’s instructions and as previously described [19]. Briefly, siRNA was complexed with 60 nmol of transfection reagent, diluted with M199/HSA to 4 ml/dish, and added to the cells for 4 h. Subsequently, 8 ml/dish of M199 growth medium was added and cells were cultured for 48 h. After this period of time, the cells were either used for biochemical analysis or for spheroid generation.

### Spheroid assay

Spheroids were prepared according to a published protocol [20]. Cells suspended in growth medium were mixed at a 4:1 ratio with methylcellulose (12 mg/ml) and 3000 cells/well were cultured in 96-well round-bottom plates for 24 h. The spheroids formed were washed with Hepes buffer and transferred to a fibrinogen solution (1.8 mg/ml in Hepes buffer) containing 20 U/ml aprotinin to give a suspension of approximately 100 spheroids per ml. 300 μl of this suspension together with 0.2 U thrombin, was added per well of a 24-well plate and incubated for 20 min at 37 °C to induce the formation of a three-dimensional fibrin gel. Thrombin was then washed out, M199 with 2% FCS containing TKI or vehicle control was added and after 30 min the cells were stimulated with 10 ng/ml VEGF. After 24 h (transfected HUVEC) or 48 h, spheroids were fixed with 4% paraformaldehyde, observed by light microscopy and photographed (AxioVert 200, Carl Zeiss, Oberkochen, Germany), and finally analysed using cellSens™ image analysis software (Olympus, Tokyo, Japan). For each experiment, the images of 5–10 spheroids per condition were used to quantify the number of sprouts per spheroid.

### Cell lysis and immunoblotting

After each treatment, cells were washed with PBS and lysed on ice in Tris buffer containing sucrose and protease inhibitors (50 mM Tris (pH 7.5), 1 mM EDTA, 1 mM EGTA, 1% (v/v) Triton X-100, 1 mM Na_3_VO_4_, 50 mM NaF, 5 mM Na_4_P_2_O_7_, 0.27 M sucrose, 0.1% (v/v) β-mercaptoethanol, 0.2 mM PMSF, 1% complete protease inhibitor cocktail). After centrifugation (13,000 × g, 10 min, 4 °C), the supernatant was collected (cell lysate) and the protein concentration was determined using Lowry reagents (DCTM Protein Assay Kit from Bio-Rad Laboratories GmbH, Feldkirchen, Germany) and bovine serum albumin (BSA) as standard.

Cell lysates (30–50 μg/lane) were supplemented with Laemmli buffer, separated by SDS-PAGE and transferred to polyvinylidene difluoride (PVDF) membranes. The membranes were blocked for 1 h in TBST buffer (20 mM Tris (pH 7.6), 137 mM NaCl, 0.1% (v/v) Tween-20) containing 5% non-fat dried skimmed milk. Membranes were incubated overnight at 4 °C with primary antibodies diluted in TBST containing 5% BSA. After incubation with the appropriate horseradish peroxidase-conjugated secondary antibodies for 1 h, signal detection was performed using an enhanced chemiluminescence reagent (ECL™ Western Blotting Detection Kit from GE Healthcare, Chicago, IL, USA). Protein bands were quantified by densitometry using ImageJ software. Ratios of phosphoprotein to total protein or ratios of protein of interest to loading control were calculated.

### Statistics

Data are presented as means ± SEM of 3–5 independent experiments. Statistical analysis was performed using Student’s *t*-test or two-way repeated measures ANOVA where applicable. ANOVA was followed by Bonferroni’s post hoc test for multiple comparisons. p < 0.05 was considered statistically significant. Statistical tests and graphs were performed using GraphPad Prism 4 software.

## Results

### Ponatinib and nilotinib differentially regulate endothelial cell angiogenesis in vitro

**Fig. 1:**
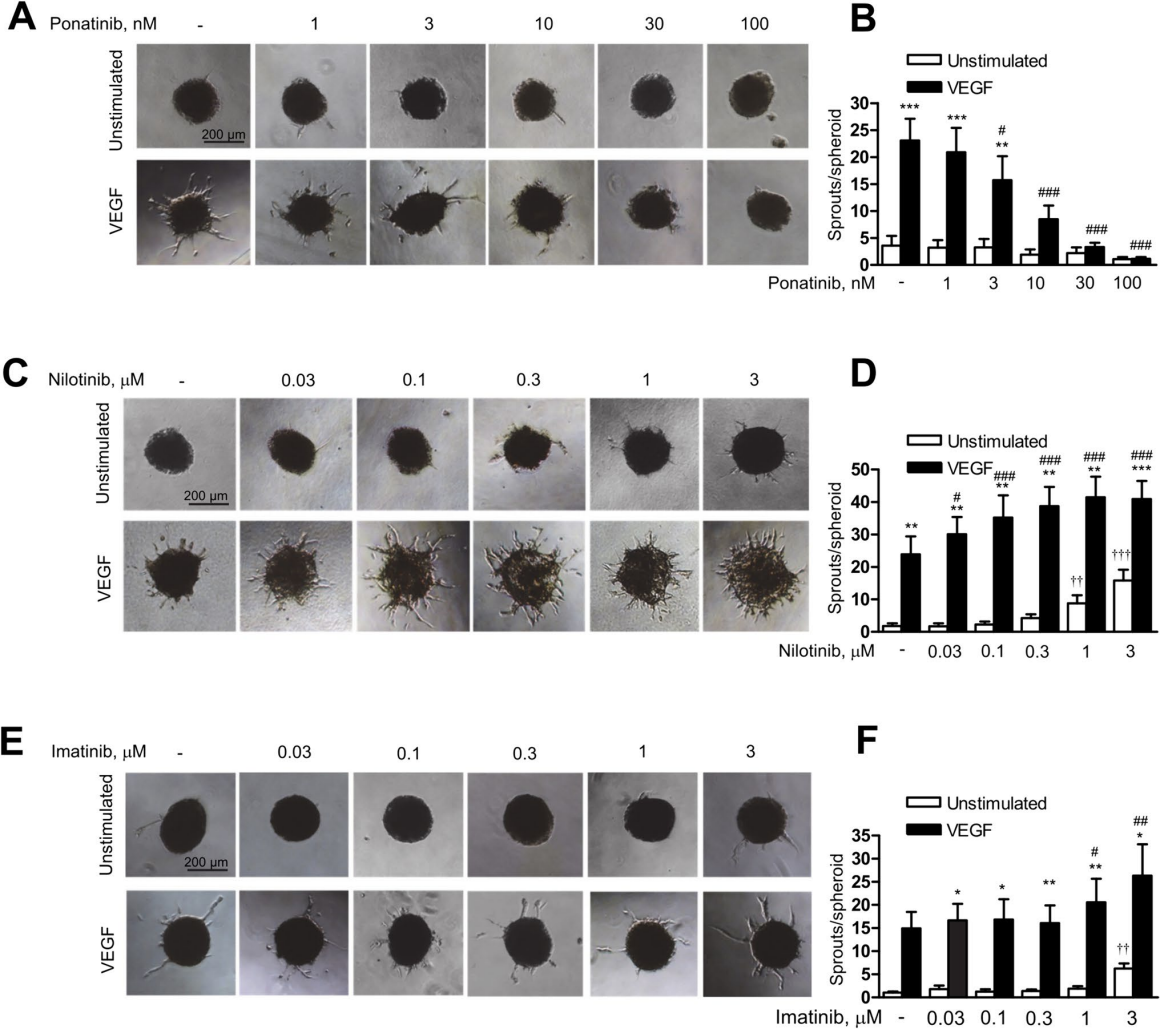
Effects of BCR::ABL1 inhibitors on angiogenesis in vitro. Human umbilical vein endothelial cells (HUVEC) spheroids were pre-treated with vehicle (-) or ponatinib (**A**–**B**), nilotinib (**C**–**D**) and imatinib (**E**–**F**) at the indicated concentrations for 30 min and then stimulated with VEGF (10 ng/ml, 48 h) or left unstimulated. Representative images (**A, C, E**) and analysis of the number of sprouts per spheroid (**B**, **D**, **F**) are shown. Data are means ± SEM of 3 (imatinib) or 4 (ponatinib and nilotinib) independent experiments using endothelial cells from different donors. *p < 0.05, **p < 0.01, ***p < 0.001 *vs*. respective unstimulated treatment; ^#^p < 0.05, ^##^p < 0.01, ^###^p < 0.001 *vs*. vehicle control at VEGF-stimulated conditions; ^††^p < 0.01, ^†††^p < 0.001 *vs.* vehicle control at unstimulated conditions

We first compared the effects of ponatinib, nilotinib and imatinib on basal and VEGF-induced angiogenesis using a spheroid assay. Endothelial spheroids were pretreated with BCR::ABL1 inhibitors at the indicated concentrations for 30 min and then stimulated with VEGF. As shown in Fig. 1A, B, ponatinib did not affect angiogenesis under basal conditions but strongly inhibited VEGF-induced sprouting in a dose-dependent manner. Significant reductions were observed with 3 nM ponatinib and a complete loss of sprouting occurred after preincubation with 100 nM ponatinib. In striking contrast to ponatinib, nilotinib significantly stimulated basal sprouting up to five and ninefold at 1 and 3 μM, respectively, and additionally potentiated VEGF-induced angiogenesis in a dose-dependent manner with a maximal effect at 1 μM (Fig. 1C, D). Imatinib had no effect on basal sprouting at concentrations below 3 µM and increased VEGF-induced sprouting only when applied at 1 or 3 µM (Fig. 1E, F).

### Inhibition of ABL kinases does not affect angiogenesis

**Fig. 2:**
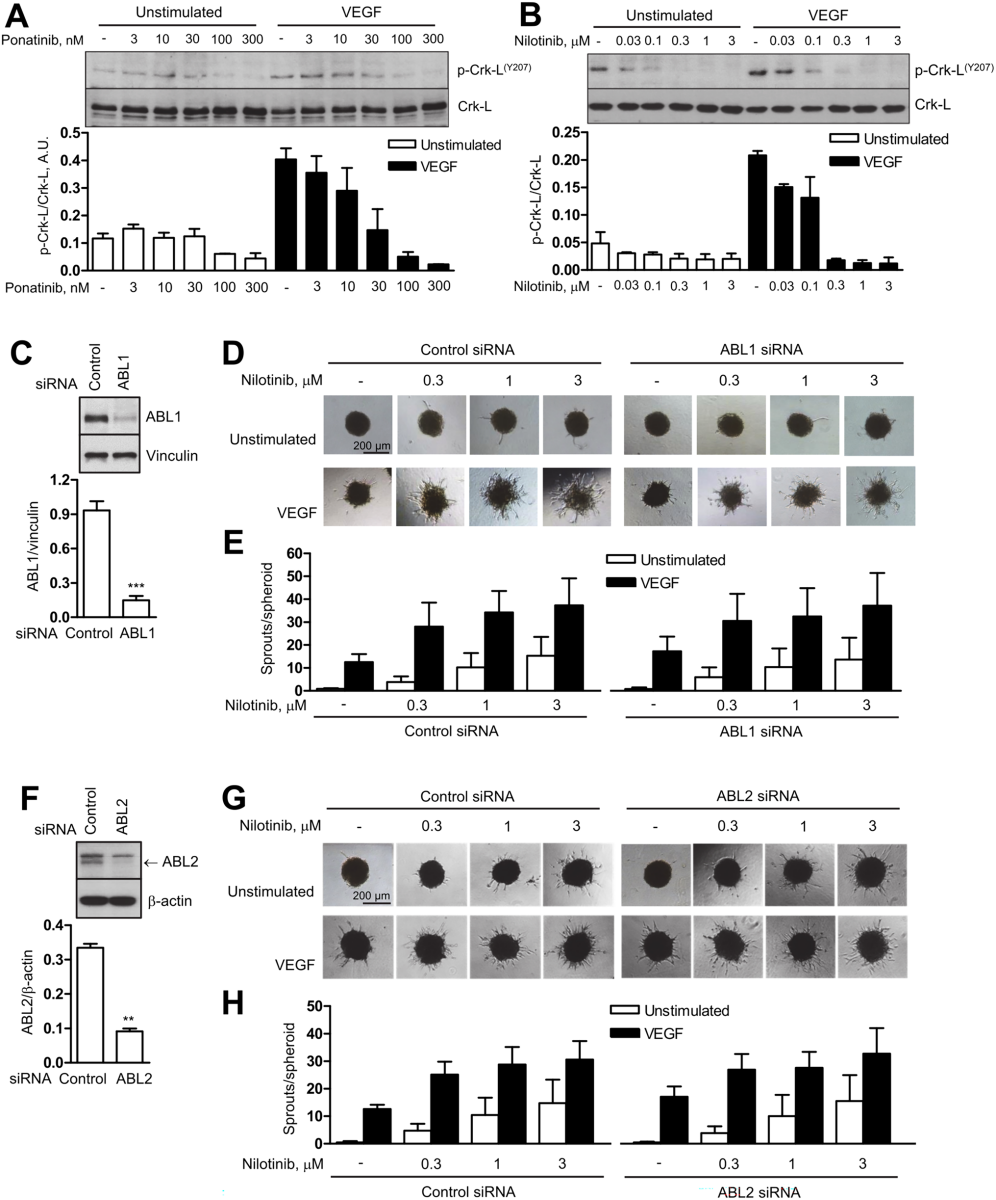
ABL kinases are not involved in effects of nilotinib on angiogenesis. Human umbilical vein endothelial cells (HUVEC) were pre-treated with vehicle (-), ponatinib (**A**) or nilotinib (**B**) at the indicated concentrations for 30 min and then stimulated with VEGF (10 ng/ml, 10 min) or left unstimulated; afterwards cells were harvested for immunoblot analysis. **C**, **F** HUVEC were transfected with indicated siRNAs for 48 h and harvested for immunoblot analysis to confirm ABL1 or ABL2 downregulation. **D**–**E**, **G**–**H** HUVEC spheroids, generated from siRNA-transfected cells, were pre-treated with vehicle (-) or nilotinib at the indicated concentrations for 30 min and then stimulated with VEGF (10 ng/ml, 24 h) or left unstimulated. Representative immunoblots (**A**–**C**, **F**) and spheroid images (**D**, **G**) with corresponding analyses (**E**, **H**) are shown. Data are means ± SEM of 3 independent experiments using endothelial cells from different donors (2 donors for Crk-L blot). **p < 0.01, ***p < 0.001 *vs*. control siRNA

ABL kinases, which have recently been described to affect endothelial survival and permeability [12, 13], are likely to be inhibited by ponatinib or nilotinib and may be involved in the adverse effects of these agents. To address this question, we first investigated whether VEGF triggers ABL signalling in endothelial cells and whether BCR::ABL1 inhibitors can interfere. We found that VEGF stimulated the phosphorylation of the ABL substrate Crk-like protein (Crk-L) at tyrosine 207, a specific ABL site (Fig. 2A, B), indicating a stimulation of the ABL pathway by VEGF. Both basal and VEGF-induced Crk-L phosphorylation were dose-dependently reduced by ponatinib or nilotinib confirming that ABL kinase is a target of both compounds in endothelial cells (Fig. 2A, B). However, ABL kinases did not affect angiogenic signalling or angiogenesis. This was demonstrated in cells, in which either ABL1 or ABL2 were downregulated by specific siRNA (Fig. 2C, F, 84% and 73% downregulation for ABL1 and ABL2, respectively). In these cells, VEGF signalling was not altered as shown by comparable phosphorylation patterns of VEGF receptor 2 (VEGFR2, serine 1175) and its downstream targets endothelial nitric oxide synthase (eNOS, serine 1177), Akt (serine 473) and Erk1/2 (threonine 202/tyrosine 204) in control and ABL-depleted cells (Supplementary Figure S1). Furthermore, neither basal nor VEGF-induced sprouting was different between cells treated with control siRNA or ABL1- or ABL2-specific siRNA (Fig. 2D–E, G–H, compare untreated conditions). We also asked whether ABL1 or ABL2 might contribute to the angiogenic effect of nilotinib. This did not appear to be the case, as nilotinib increased both basal and VEGF-induced sprouting to a similar extent in both control and ABL1-or ABL2-depleted cells (Fig. 2D–E, G–H).

### The angiogenic effect of nilotinib is not dependent on DDR1 kinase

**Fig. 3:**
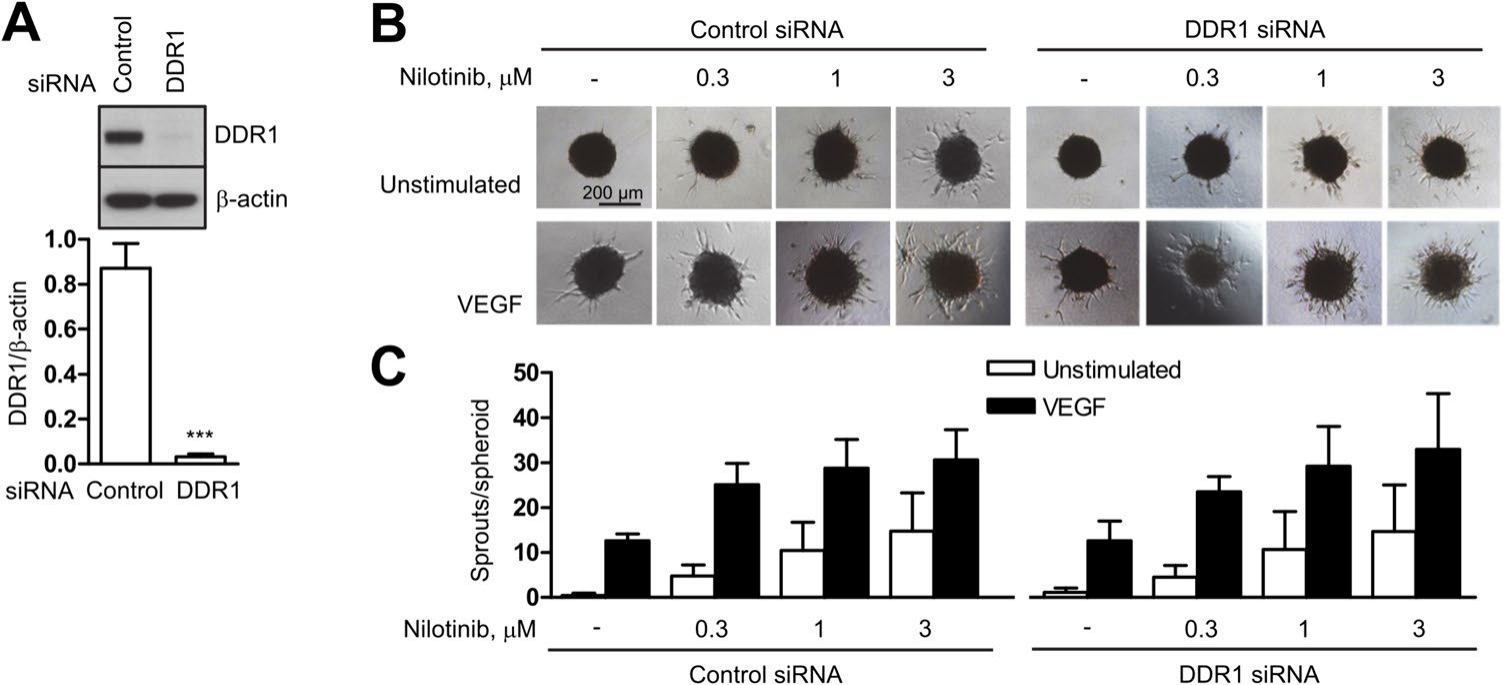
DDR1 is not involved in the effects of nilotinib on angiogenesis. **A** Human umbilical vein endothelial cells (HUVEC) were transfected with DDR1 siRNA for 48 h and harvested for immunoblot analysis to confirm DDR1 downregulation. **B**, **C** HUVEC spheroids, generated from DDR1 siRNA-transfected cells, were pre-treated with vehicle (-) or nilotinib at the indicated concentrations for 30 min and then stimulated with VEGF (10 ng/ml, 24 h) or left unstimulated. Representative immunoblot (**A**) and spheroid images (**B**) with corresponding analysis (**C**) are shown. Data are means ± SEM of 3 independent experiments using endothelial cells from different donors

We next investigated a potential role for DDR1 in nilotinib-induced sprouting. DDR1 is a tyrosine kinase receptor that binds fibrillar collagen and has been reported to be a target of nilotinib [9]. We efficiently downregulated DDR1 by approximately 96% using an siRNA approach (Fig. 3A) und examined the angiogenic effect of nilotinib in DDR1-expressing and DDR1-depleted cells. Figure 3B, C show that nilotinib-induced angiogenesis was not altered by DDR1 downregulation. Taken together, the data described here and in the previous section suggest that the angiogenic effects of nilotinib are most likely due to other off-target mechanism(s), rather than to its targets ABL1/2 or DDR1.

### Imatinib has no effect on VEGF-induced angiogenic signalling

**Fig. 4:**
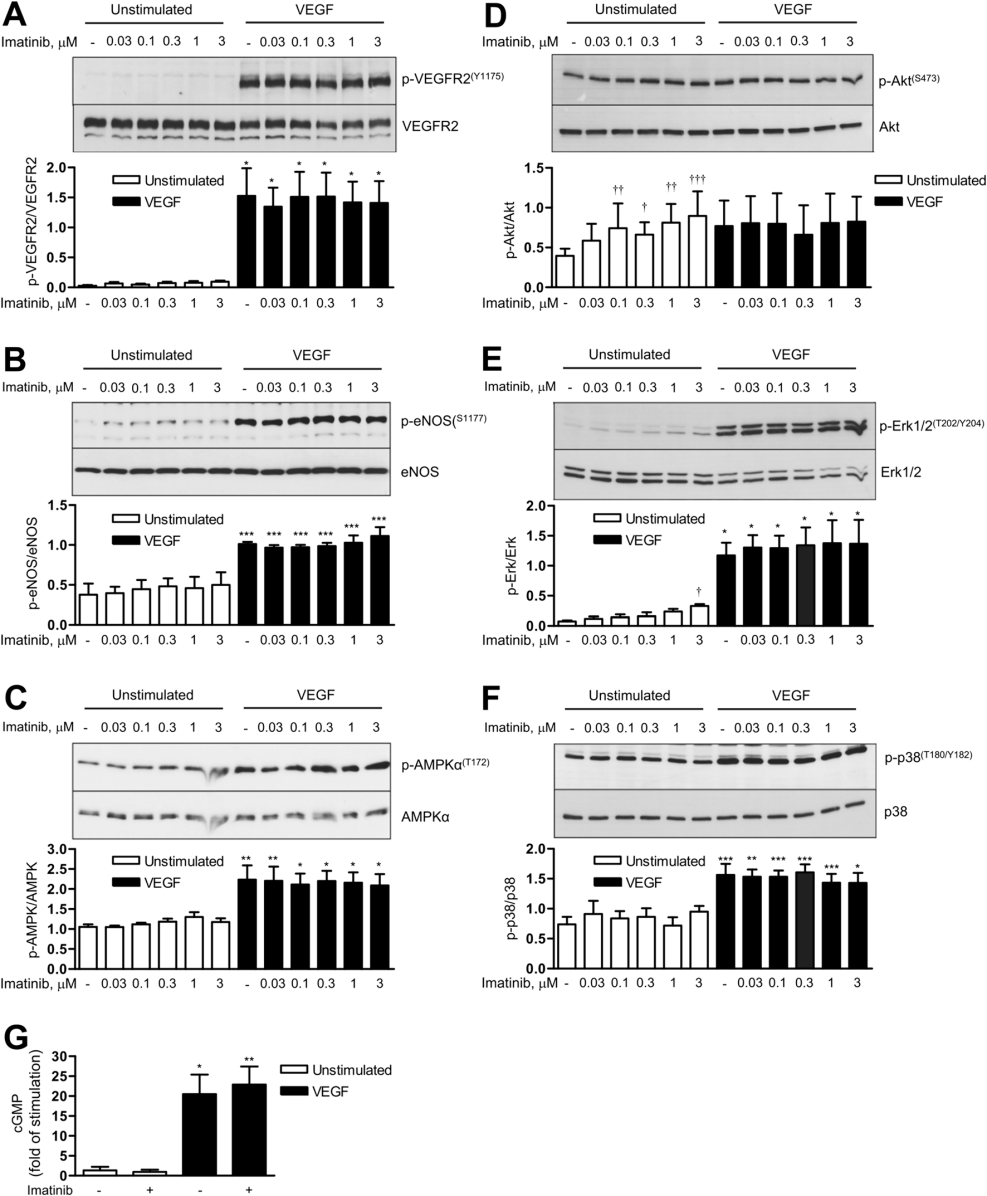
Effects of imatinib on VEGF signalling and cGMP production. Human umbilical vein endothelial cells (HUVEC) were pre-treated with vehicle (-) or imatinib at the indicated concentrations for 30 min, then stimulated with VEGF (50 ng/ml) for 5 min (**A**–**C**) or 10 min (**D**–**F**) or left unstimulated for the corresponding periods of time; afterwards cells were harvested for immunoblot analysis. Representative immunoblots and densitometric analyses are shown. **G** HUVEC monolayers were pre-incubated with vehicle (-) or imatinib at the plateau concentration (3 µM) for 30 min, then stimulated with VEGF (50 ng/ml, 20 min) or left unstimulated. Afterwards, cell lysates were used for determination of cGMP levels using an ELISA kit. Data are means ± SEM of 3 independent experiments using endothelial cells from different donors. *p < 0.05, **p < 0.01, ***p < 0.001 *vs*. respective unstimulated treatment; ^†^p < 0.05, ^††^p < 0.01, ^†††^p < 0.05 *vs.* vehicle control at unstimulated conditions

Our next approach was to investigate the effect of BCR::ABL1 inhibitors on VEGF-induced angiogenic signalling pathways as monitored by VEGF-induced phosphorylation of VEGFR2 and its downstream targets. Imatinib had no effect on basal or VEGF-induced phosphorylation of VEGFR2, eNOS, AMPK and p38 kinase (Figure 4A–C, F) and did not alter VEGF-induced cGMP generation (Fig. 4G), an indicator of endogenous NO formation. At higher concentrations, a moderate increase in phosphorylation of Akt (serine 473) and Erk1/2 (threonine 202/tyrosine 204) was observed (Fig. 4D, E).

### Ponatinib inhibits VEGF-induced angiogenic signalling

**Fig. 5:**
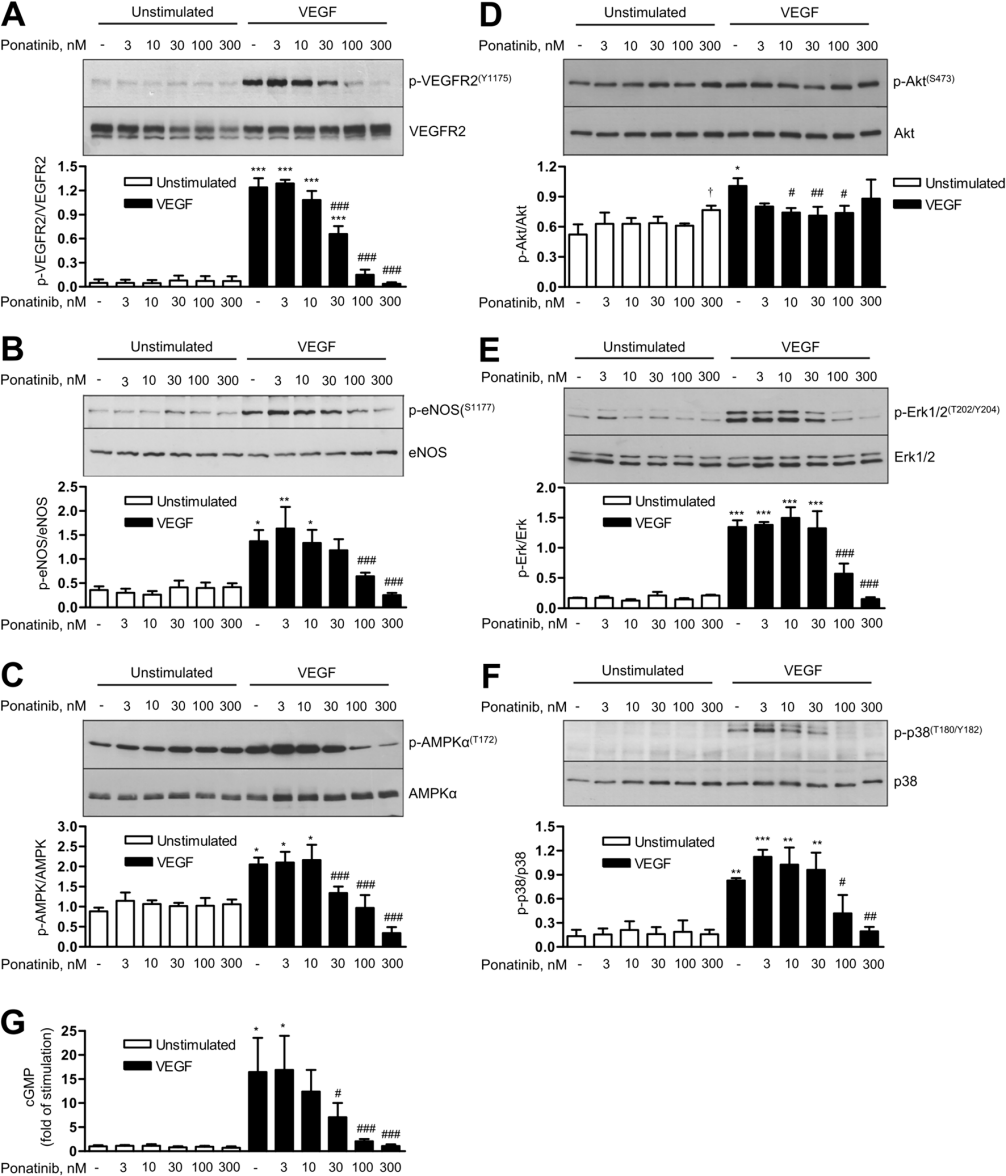
Effects of ponatinib on VEGF signalling and cGMP production. Human umbilical vein endothelial cells (HUVEC) were pre-treated with vehicle (-) or ponatinib at the indicated concentration for 30 min, then stimulated with VEGF (50 ng/ml) for 5 min (**A**–**C**) or 10 min (**D**–**F**) or left unstimulated for the corresponding periods of time. Afterwards, cells were harvested for immunoblot analysis. Representative immunoblots and densitometric analyses are shown. **G** HUVEC monolayers were pre-incubated with vehicle (-) or ponatinib at the indicated concentrations for 30 min, then stimulated with VEGF (50 ng/ml, 20 min) or left unstimulated. Afterwards, cell lysates were used for determination of cGMP levels using ELISA kit. Data are means ± SEM of 3–4 independent experiments using endothelial cells from different donors (2 donors for phospho-and total p38 blots). *p < 0.05, **p < 0.01, ***p < 0.001 *vs*. respective unstimulated treatment; ^#^p < 0.05, ^##^p < 0.01, ^###^p < 0.001 *vs*. vehicle control at VEGF-stimulated conditions; ^†^p < 0.05 *vs.* vehicle control at unstimulated conditions

Ponatinib caused a dose-dependent inhibition of VEGFR2 phosphorylation at serine 1175 that was significant at 30 nM, and complete at 100 nM (Fig. 5A). Correspondingly, phosphorylation of proteins downstream of VEGFR2, including eNOS (serine 1177), AMPK (threonine 172), Erk1/2 (threonine 202/tyrosine 204) and p38 kinase (threonine 180/tyrosine 182) showed significant reductions (Fig. 5B, C, E, F), whereas phosphorylation of Akt (serine 473) was little affected (Fig. 5D). In parallel with the inhibition of eNOS activation, the nitric oxide (NO)/cGMP pathway was suppressed by ponatinib as shown by the dose-dependent reduction of VEGF-induced cGMP levels (Fig. 5G). Taken together and in agreement with a previous study [14], these data demonstrate that ponatinib is an effective and potent inhibitor of VEGF signalling.

### Nilotinib triggers angiogenic signalling and potentiates VEGF-induced effects

**Fig. 6:**
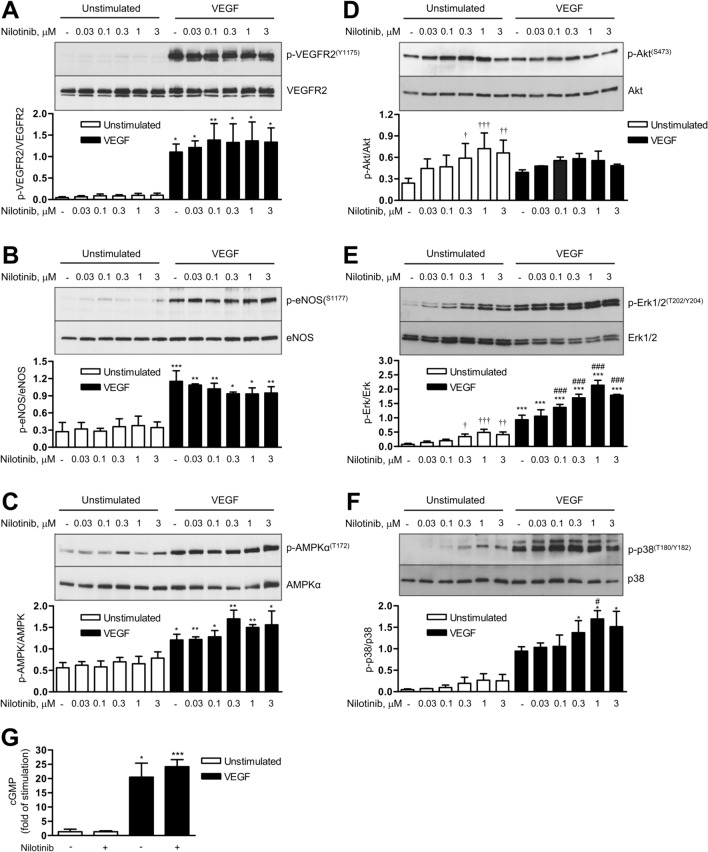
Effects of nilotinib on VEGF signalling and cGMP production. Human umbilical vein endothelial cells (HUVEC) were pre-treated with vehicle (-) or nilotinib at the indicated concentrations for 30 min, then stimulated with VEGF (50 ng/ml) for 5 min (**A**–**C**) or 10 min (**D**–**F**) or left unstimulated for the corresponding periods of time; afterwards, cells were harvested for immunoblot analysis. Representative immunoblots and densitometric analysis are shown. **G** HUVEC monolayers were pre-incubated with vehicle (-) or nilotinib at the plateau concentration (3 µM) for 30 min, then stimulated with VEGF (50 ng/ml, 20 min) or left unstimulated. Afterwards, cell lysates were used for determination of cGMP levels using an ELISA kit. Data are means ± SEM of 3 independent experiments using endothelial cells from different donors (2 donors for phospho-and total p38 blots). *p < 0.05, **p < 0.01, ***p < 0.001 *vs*. respective unstimulated treatment; ^#^p < 0.05, ^###^p < 0.001 *vs*. vehicle control at VEGF-stimulated conditions; ^†^p < 0.05, ^††^p < 0.05, ^†††^p < 0.05 *vs.* vehicle control at unstimulated conditions

In contrast to ponatinib, nilotinib did not inhibit phosphorylation of VEGFR2 or its downstream targets eNOS, AMPK, Akt, Erk1/2 or p38 kinase (Fig. 6A–F) and had no effect on VEGF-induced cGMP generation (Fig. 6G). However, nilotinib significantly stimulated the phosphorylation of several angiogenic proteins in a dose-dependent manner under basal and/or VEGF-stimulated conditions. Specifically, nilotinib increased basal Akt phosphorylation at serine 473 up to threefold and Erk1/2 phosphorylation at threonine 202/tyrosine 204 up to fivefold (Fig. 6D, E). In addition, VEGF-induced phosphorylation of Erk1/2 and p38 kinase was increased by approximately twofold by nilotinib (Fig. 6E, F).The effects of nilotinib on basal angiogenesis were achieved by bypassing VEGFR2 activation as no autophosphorylation of VEGFR2 at serine 1175 was observed (Fig. 6A). We also excluded the involvement of VEGFR1, a negative regulator of VEGFR2 signalling, in the angiogenic effects of nilotinib, as siRNA-mediated downregulation of VEGFR1 did not alter nilotinib-induced sprouting (Supplementary Figure S2).

Taken together, these data suggest that nilotinib stimulates angiogenesis through pathways involving Akt, Erk1/2 or p38 but most likely not involving interaction with VEGF receptors.

### Erk1/2 signalling mediates the angiogenic effect of nilotinib

**Fig. 7:**
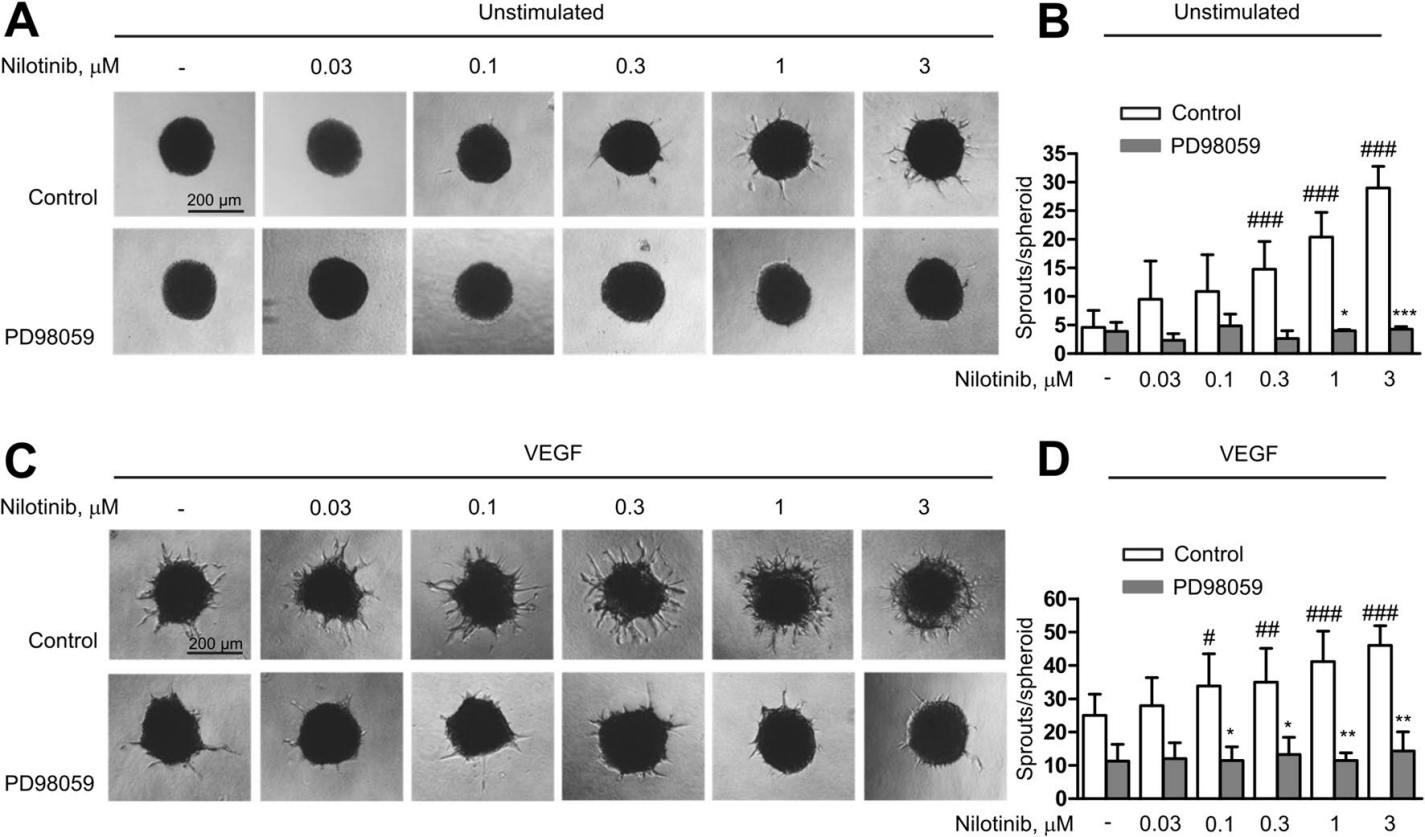
Erk1/2 mediates pro-angiogenic effect of nilotinib. Human umbilical vein endothelial cells** (**HUVEC) spheroids were pre-treated with either PD98056 (20 μM, 30 min) or solvent (control) prior to treatment with vehicle (-) or nilotinib at the indicated concentrations for 30 min. Afterwards, spheroids were left either unstimulated (**A**–**B**) or were stimulated with VEGF (10 ng/ml, 48 h) (**C**–**D**) Representative images (**A**, **C**) and analyses of the number of sprouts per spheroid (**B**, **D**) are shown. Data are mean ± SEM of 3 independent experiments using endothelial cells from different donors. *p < 0.05, **p < 0.01, ***p < 0.001 *vs*. respective control treatment; ^#^p < 0.05, ^##^p < 0.01, ^###^p < 0.001 *vs*. vehicle (-) control

Since nilotinib most strongly promoted the phosphorylation of Erk1/2, an essential angiogenic protein, we investigated whether the pro-angiogenic effect of nilotinib was due to Erk1/2 signalling. To this end, we used PD98059, a specific inhibitor of the Erk1/2 upstream kinases MEK1/2 [21]. As shown in Fig. 7A–D, PD98059 completely prevented nilotinib-induced sprouting and nilotinib was unable to rescue the PD98059-mediated impairment of VEGF-induced angiogenesis. In contrast, suppression of p38 with BIRB796, an inhibitor of all p38 MAPK isoforms [22], did not inhibit nilotinib-induced sprouting under basal or VEGF-stimulated conditions, but actually stimulated basal angiogenesis and potentiated the proangiogenic effect of nilotinib (Supplementary Figure S3A–D). These findings confirm the essential role of Erk1/2 signalling in mediating the effects of nilotinib on angiogenesis.

### Fyn-related kinase (FRK), a potential off-target of nilotinib inhibits endothelial EGFR and angiogenesis

To further elucidate the pathway leading to Erk1/2 phosphorylation by nilotinib, we searched for a target kinase that could negatively regulate Erk signalling. We used publicly available screening data of nilotinib tested against 442 kinases in a competition binding assay using the KINOMEscan® assay platform, Eurofins DiscoverX (HMS LINCS data collection, http://lincs.hms.harvard.edu/kinomescan/). Based on criteria such as low Kd value, nilotinib Kd value *vs.* imatinib Kd value, and biological functions described in the literature, we proposed FRK as a potential nilotinib off-target involved in its angiogenic effects. FRK has been shown to act as a negative regulator of epidermal growth factor receptor (EGFR) signalling involving Erk1/2 activation [23].

**Fig. 8:**
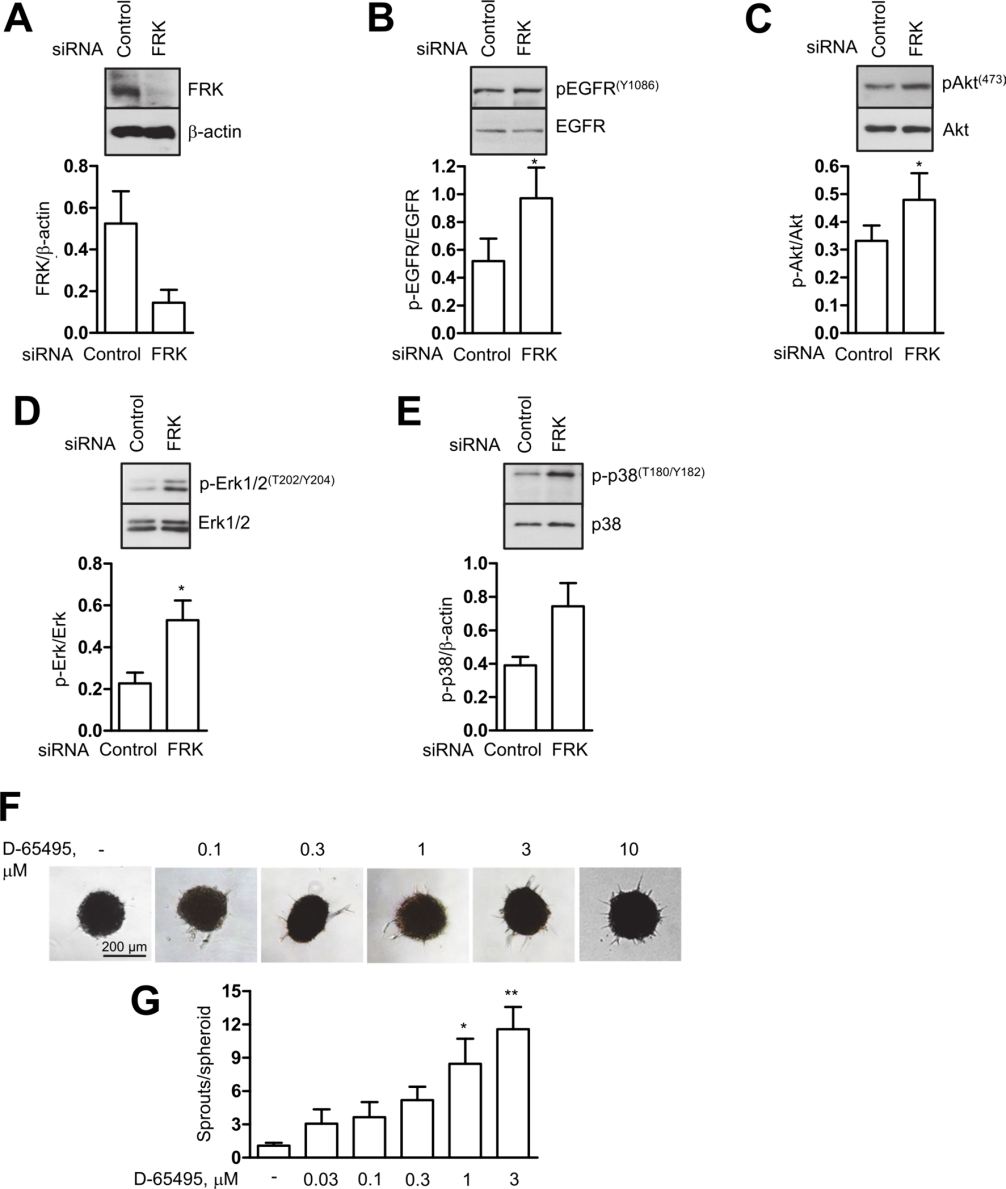
FRK inhibition activates EGFR signalling and increases angiogenesis. Human umbilical vein endothelial cells (HUVEC) were transfected with FRK siRNA for 24 h and harvested for immunoblot analysis to confirm FRK downregulation (**A**) as well as phosphorylation states of components of EGFR signalling (**B**–**D**). **F**, **G** HUVEC spheroids were pre-treated with vehicle (-) or D-65495 at the indicated concentrations for 30 min and then stimulated with VEGF (10 ng/ml, 48 h) or left unstimulated. Representative immunoblots (**A**–**E**) and spheroid images (**F**) with corresponding analyses (**G**) are shown. Data are means ± SEM of 3 independent experiments using endothelial cells from different donors. *p < 0.05, **p < 0.01*vs*. control

To test whether an inhibition of FRK could activate Erk1/2 phosphorylation via EGFR in endothelial cells, we downregulated FRK using an siRNA approach and examined phosphorylation events in the EGFR pathway. As shown in Fig. 8A–E, we found an increased phosphorylation of EGFR at tyrosine 1086 as well as phosphorylation of Akt, p38 and Erk1/2 suggesting that inhibition of FRK promotes EGFR signalling even in the absence of added ligand, i.e. epidermal growth factor (EGF). Consistent with this, the FRK inhibitor D-65495 [24] had a pro-angiogenic effect similar to that of nilotinib (Fig. 8F, G).

Taken together, these data confirm that FRK negatively regulates EGFR signalling in endothelial cells and that its inhibition potentiates angiogenesis. Thus, FRK may be a potential target of nilotinib mediating its angiogenic effects.

### The EGFR pathway is involved in the angiogenic effect of nilotinib

**Fig. 9:**
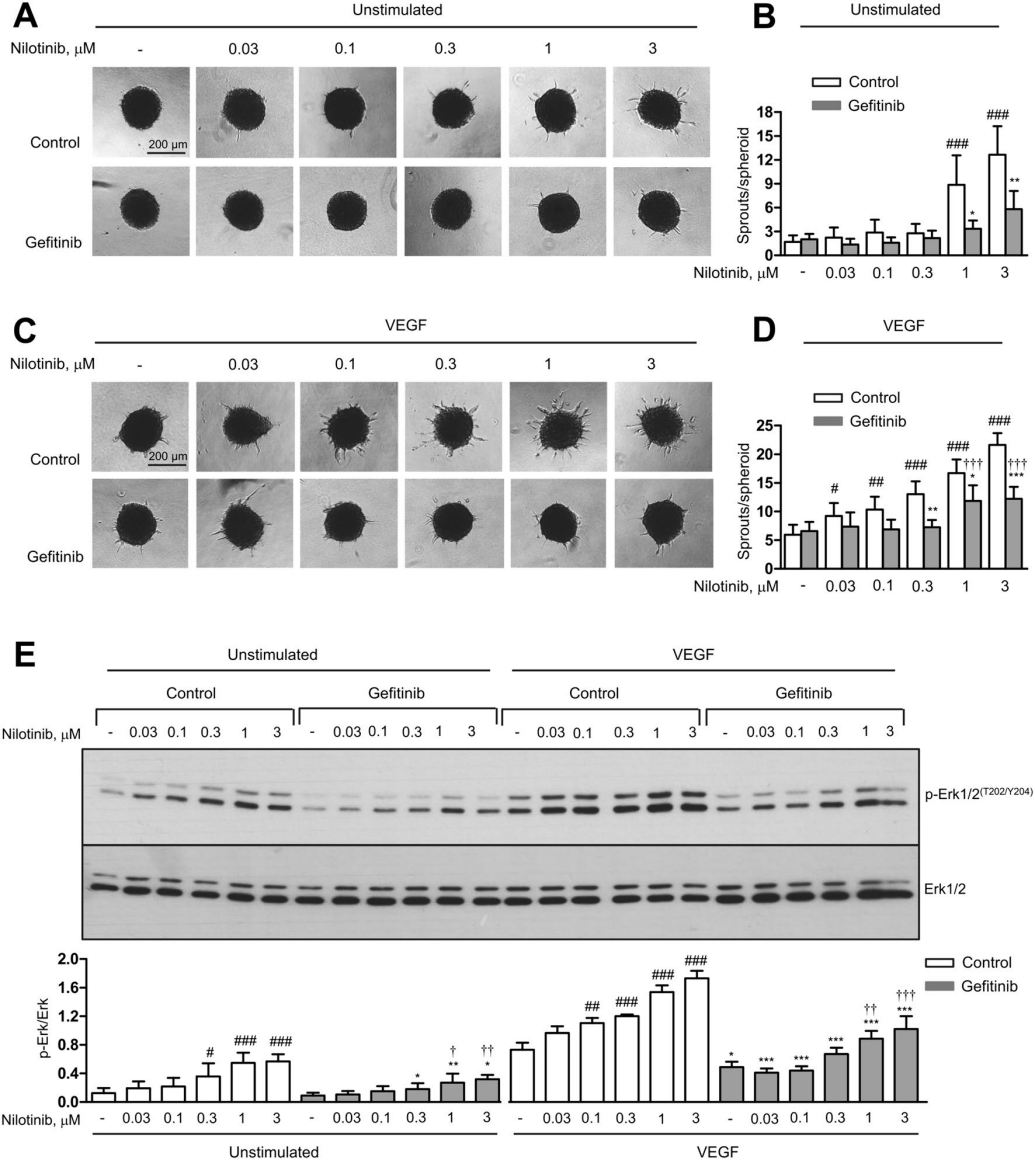
Gefitinib decreases the effects of nilotinib on angiogenesis and Erk1/2 phosphorylation. Human umbilical vein endothelial cells (HUVEC) spheroids were pre-treated with gefitinib (3 μM, 30 min) or solvent (control) prior to treatment with vehicle (-) or nilotinib at the indicated concentrations for 30 min. Afterwards, spheroids were left either unstimulated (**A**–**B**) or were stimulated with VEGF (10 ng/ml, 24 h) (**C**–**D**). **E** HUVEC were pre-treated with gefitinib (1 µM, 30 min) or solvent (control) prior to treatment with vehicle (-) or nilotinib at the indicated concentrations for 30 min. Afterwards, cells were stimulated with VEGF (50 ng/ml, 10 min) or left unstimulated and, finally, harvested for immunoblot analysis. Representative images (**A**, **C**, **E**) and corresponding analyses (**B**, **D**) are shown. Data are means ± SEM of independent experiments using endothelial cells from 4 (immunoblot) and 5 (spheroid assay) different donors. *p < 0.05, **p < 0.01, ***p < 0.001 *vs*. respective control treatment; ^#^p < 0.05, ^##^p < 0.01, ^###^p < 0.001 *vs*. vehicle (-) at control treatment; ^†^p < 0.05, ^††^p < 0.01, ^†††^p < 0.001 *vs.* vehicle (-) at gefitinib treatment

To address the hypothesis that nilotinib may lead to activation of the EGFR pathway via inhibiting FRK, we used the EGFR inhibitor gefitinib, which selectively targets EGFR and does not inhibit VEGF signalling in our study (Supplementary Figure S4A–F). Pretreatment of endothelial cells with gefitinib significantly reduced the pro-angiogenic effect of nilotinib under both basal and VEGF-stimulated conditions (Fig. 9A–D). In addition, gefitinib significantly decreased nilotinib-induced Erk1/2 phosphorylation (Fig. 9E). These data suggest that EGFR signalling may be involved in mediating the angiogenic effects of nilotinib and that the relief of EGFR inhibition by targeting FRK might be the underlying mechanism.

## Discussion

Vascular side effects associated with the use of BCR::ABL1 inhibitors, particularly with ponatinib and nilotinib [25–28], have emerged as a major concern in the management of CML. The mechanisms underlying these side effects are poorly understood but may involve alterations in the vascular endothelium. In this study, we investigated the effects of three BCR::ABL1 TKIs, namely ponatinib, nilotinib and imatinib, on basal and VEGF-induced endothelial angiogenesis and angiogenic signalling pathways in vitro. Angiogenesis, a major function of endothelial cells, is a multi-step process involving degradation of the extracellular matrix, migration and proliferation of endothelial cells and the formation of tube-like structures that eventually develop into mature vessels to allow blood flow and the delivery of oxygen and nutrients to the tissues. Parts of this process are reflected in the spheroid model used in our study, in which endothelial spheroids were embedded in a fibrin matrix and stimulated with VEGF and the resulting sprouting was quantified as a measure of angiogenic capacity. The TKIs tested were used in a pharmacological concentration range with the highest concentration corresponding to the peak level of the compound in the patient's blood [14, 15, 29–31]. The data obtained in this study show that ponatinib inhibited VEGF-induced angiogenesis in vitro, which was due to inhibition of VEGFR2-mediated signalling events. In contrast, nilotinib had pro-angiogenic effects, possibly involving the EGFR signalling pathway. These opposing effects on angiogenesis may contribute to adverse events in the vasculature and may need to be considered in TKI treatment regimens in patients at risk of vascular disease.

The anti-angiogenic effect of ponatinib has previously been observed in vitro as a reduced ability of endothelial cells to form tubes on a Matrigel matrix in the absence of any stimulus [14] and in vivo using the zebrafish model [32]. In contrast, there are conflicting data for nilotinib showing either no effect on spontaneous tube formation [14] or inhibition of VEGF-induced tube formation [17], which may be related to the experimental settings and doses of nilotinib used. Nilotinib has also been shown to inhibit basal and VEGF-induced endothelial proliferation and migration [16, 17], to induce an inflammatory phenotype in endothelial cells and to reduce vessel density in a mouse model of hindlimb ischemia [17]. However, the pro-angiogenic effect of nilotinib described in our study at pharmacologically relevant concentrations is a novel observation.

The pro-angiogenic effect of nilotinib was not due to inhibition of endogenous ABL1 or ABL2 kinases. These kinases have been described to modulate Tie2 expression and angiopoietin-1-mediated endothelial cell survival and their loss results in vascular dysfunction and tissue damage [12, 13]. VEGF has been shown to stimulate these kinases [12], which was confirmed in our study by showing that the ABL-specific phosphorylation site on Crk-L (tyrosine 207) was phosphorylated by VEGF [33]. Furthermore, endothelial ABL1 and ABL2 kinases were targeted by TKIs in our study, as ponatinib and nilotinib inhibited Crk-L phosphorylation in a dose-dependent manner. However, siRNA-mediated downregulation of either ABL1 or ABL2 did not alter the pro-angiogenic effect of nilotinib suggesting that it is independent of ABL kinases. Similarly, DDR1, another known target of nilotinib was not involved in stimulating angiogenesis, as nilotinib induced sprouting even when DDR1 was downregulated in endothelial cells with specific siRNA.

To further elucidate the mechanisms underlying the anti- or proangiogenic effects of TKIs, we performed signalling studies in TKI-pretreated endothelial cells and followed the phosphorylation kinetics of VEGFR2 and its downstream targets in response to VEGF. Imatinib had almost no effect on VEGF-induced signalling pathways and increased basal Akt and Erk1/2 phosphorylation only at high concentrations (3 µM), which may explain the observed increase in basal angiogenesis under these conditions. Ponatinib treatment of cells resulted in a dose-dependent inhibition of VEGFR2 phosphorylation at serine 1175 in response to VEGF indicating reduced receptor activation. Accordingly, signalling events downstream of VEGFR2 such as VEGF-induced phosphorylation of eNOS, AMPK, Erk 1/2 and p38 kinase and VEGF-induced cGMP generation, all of which contribute to angiogenic processes [34–37], were inhibited by ponatinib, likely explaining its inhibitory effect on angiogenesis. Thus, our data confirm VEGFR2 as a potential target of ponatinib and are consistent with a previous study in which VEGFR2 overexpression rescued the inhibitory effect of ponatinib on HUVEC tube formation [14]. In contrast to ponatinib, nilotinib did not inhibit VEGF-induced angiogenic signalling, but itself induced the phosphorylation of several angiogenic signalling molecules such as Akt, Erk1/2 and p38 and enhanced the VEGF-induced phosphorylation of Akt and Erk1/2. Accordingly, the pro-angiogenic effect of nilotinib observed in our study was related to the activation or potentiation of known angiogenic signalling pathways. In line with this, activation of the Erk1/2 and Akt pathways by nilotinib has also been reported in C2C12 myoblasts [38]. Furthermore, activation of Erk1/2 was observed in CML cell lines and primary CML patient cells treated with nilotinib in vitro [39].

Phosphorylation of Erk1/2 in response to nilotinib appears to be an important event in nilotinib-induced angiogenesis. It was the most pronounced response to nilotinib and, importantly, its prevention by pharmacological inhibition of the Erk1/2 upstream kinases MEK1/2 abolished the angiogenic effect of nilotinib. This raised the question of how nilotinib leads to Erk1/2 phosphorylation. We thought that because of its mechanism of action, nilotinib might inhibit a repressive pathway rather than have a direct activating effect on Erk1/2. In addition, the putative target should be specific to nilotinib but not to imatinib. Based on our search in the KINOMEscan assay platform provided by Eurofins/DiscoverX (http://lincs.hms.harvard.edu/db/datasets/20162/results), FRK, a non-receptor tyrosine kinase and member of the BRK family kinases (reviewed in [40]), was selected as a possible candidate kinase out of 442 kinases against which nilotinib was screened in a completion binding assay. FRK has been shown to act as a growth inhibitor in several cancer cell lines, in part by inhibiting signalling pathways such as JAK/STAT, MAPK, Akt and the EGFR pathway [23, 41–44]. Our data confirm that FRK, which has been little studied in endothelial cells, acts as a negative regulator of tonic EGFR signalling in endothelial cells, possibly mediated by autocrine mechanisms, and, consequently, as an inhibitor of angiogenesis. Based on these findings, we hypothesize that inhibition of FRK by nilotinib may activate angiogenic signalling, including phosphorylation of Erk1/2. Consistent with this, our data show that EGFR pathway is involved in the pro-angiogenic effects of nilotinib, as both nilotinib-induced angiogenesis and Erk1/2 phosphorylation were significantly inhibited by gefitinib, a specific EGFR inhibitor. Although further experiments are needed to define the precise role of FRK as a direct endothelial target of nilotinib, our data provide the first mechanistic evidence that the FRK-EGFR axis mediates the angiogenic effects of nilotinib.

To date, adverse vascular side effects of ponatinib or nilotinib have not been associated with angiogenesis and, to our knowledge, there are no reports on the effects of TKI treatment on angiogenesis in CML patients. However, a decreased angiogenesis and suppression of VEGF signalling caused by ponatinib may be a risk factor for cardiotoxicity as it has been postulated for VEGF inhibitor therapy (reviewed in [45]). In contrast, increased angiogenesis as seen with nilotinib may aggravate comorbidities with pathological angiogenesis as described in arthritis, obesity and ocular diseases or initiate them in at-risk patients [46]. Thus, the results of this study may help to guide decisions about individualised therapy in CML patients, particularly in those with ocular manifestations, in which angiogenesis may play a role [47]. However, as our observations were made in cell culture experiments, these findings will need to be substantiated in further experimental and clinical studies.

## Conclusions

This study shows that ponatinib and nilotinib alter VEGF-induced angiogenesis and angiogenic signalling in opposite ways while imatinib has no effect. Ponatinib inhibits VEGF-induced angiogenesis through inhibition of VEGFR2 signalling whereas nilotinib has proangiogenic effects, probably through activation of EGFR signalling via inhibiting its negative regulator FRK. The described molecular mechanisms may contribute to the vascular side effects of these agents, which must be taken into account when making therapeutic decisions. Our data suggest that screening new compounds for CML treatment for their angiogenic side effects may help to generate drugs with a better risk–benefit profile.

## Supplementary Information

Below is the link to the electronic supplementary material.

Supplementary file1 (JPG 1422 KB)—ABL kinases do not modulate VEGF signalling. Human umbilical vein endothelial cells (HUVEC) were transfected either with ABL-1 (A–D) or ABL-2 siRNA (E–H) for 48 h and then stimulated with VEGF (50 ng/ml) for the indicated periods of time; afterwards cells were harvested for immunoblot analysis. Representative immunoblots and densitomentric analyses are shown. Data are mean ± SEM of 2 independent experiments using endothelial cells from different donors

Supplementary file2 (JPG 757 KB)—(A) Human umbilical vein endothelial cells (HUVEC) were transfected with VEGFR1 siRNA for 48 h and harvested for immunoblot analysis to confirm VEGFR1 downregulation. (B, C) HUVEC spheroids, generated from VEGFR1 siRNA-transfected cells, were pre-treated with vehicle (-) or nilotinib at the indicated concentrations for 30 min and then stimulated with VEGF (10 ng/ml, 24 h) or left unstimulated. Representative immunoblot (A) and spheroid images (B) with corresponding analysis (C) are shown. Data are means ± SEM of 3 independent experiments using endothelial cells from different donors. *p<0.05, **p<0.01, ***p<0.001 vs. vehicle (-) treatment in cells transfected with control siRNA; ###p<0.001 vs. vehicle (-) treatment in cells transfected with VEGFR1 siRNA; †p<0.05, †††p<0.001 vs. control siRNA at the corresponding condition

Supplementary file3 (JPG 1224 KB)—Pan-p38 MAPK inhibitor does not affect the pro-angiogenic effect of nilotinib. Human umbilical vein endothelial cells (HUVEC) spheroids were pre-treated with either BIRB796 (10 μM, 30 min) or solvent (control) prior to treatment with vehicle (-) or nilotinib at the indicated concentrations for 30 min. Afterwards, spheroids were left either unstimulated (A–B) or were stimulated with VEGF (10 ng/ml, 24 h) (C–D). Representative images (A, C) and analyses of the number of sprouts per spheroid (B, D) are shown. Data are mean ± SEM of 3 independent experiments using endothelial cells from different donors. *p<0.05 vs. respective control; #p<0.05, ##p<0.01 ###p<0.001 vs. vehicle (-) treatment under control conditions, †††p<0.001 vs. vehicle (-) treatment under BIRB796 treatment conditions

Supplementary file4 (JPG 1077 KB)—Gefitinib inhibits EGFR but not VEGF signalling. Human umbilical vein endothelial cells (HUVEC) were pre-treated with gefitinib (1 μM, 30 min) or vehicle (control) prior to stimulation with either VEGF (50 ng/ml) (A–C) or EGFR (100 ng/ml) (D–F) for the indicated time points; afterwards cells were harvested for immunoblot analysis. Representative images and densitometric analyses are shown. Data are mean ± SEM of 2 independent experiments using endothelial cells from different donors

## Data Availability

The representative data of all datasets generated during the current study are included in this published article (and its supplementary information files). All raw data are available from the corresponding author upon reasonable request.

## Abbreviations

AMPK: AMP-activated protein kinase
ABL1: Abelson murine leukaemia viral oncogene homolog 1
BCR: Breakpoint cluster region protein
CML: Chronic myeloid leukaemia
DDR1: Discoidin domain receptor 1
EGFR: Epidermal growth factor receptor
eNOS: Endothelial nitric oxide synthase
ERK: Extracellular signal-regulated kinase
FRK: Fyn-related kinase
HSA: Human serum albumin
HUVEC: Human umbilical vein endothelial cells
TKI: Tyrosine kinase inhibitors
VEGF: Vascular endothelial growth factor
VEGFR: Vascular endothelial growth factor receptor
