# Supplementary figures and images for: The BCR::ABL1 tyrosine kinase inhibitors ponatinib and nilotinib differentially affect endothelial angiogenesis and signalling
Source: Mol Cell Biochem. 2024 Jul 15;480(3):1627–43. doi: 10.1007/s11010-024-05070-5 (PMC11842422; doi:10.1007/s11010-024-05070-5)

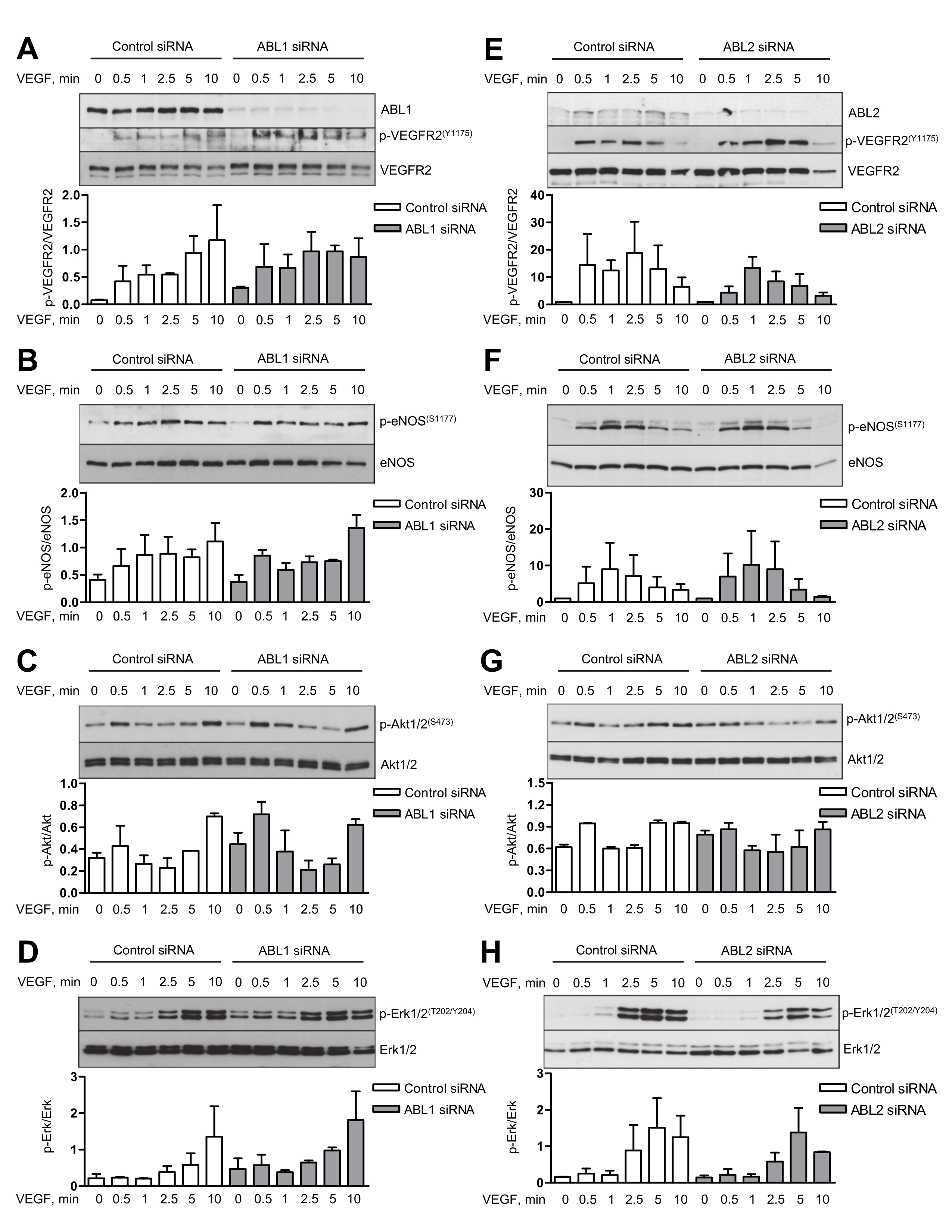

Supplement: Supplementary file 1 — Supplementary file1 (JPG 1422 KB)—ABL kinases do not modulate VEGF signalling. Human umbilical vein endothelial cells (HUVEC) were transfected either with ABL-1 (A–D) or ABL-2 siRNA (E–H) for 48 h and then stimulated with VEGF (50 ng/ml) for the indicated periods of time; afterwards cells were harvested for immunoblot analysis. Representative immunoblots and densitomentric analyses are shown. Data are mean ± SEM of 2 independent experiments using endothelial cells from different donors [file 11010_2024_5070_MOESM1_ESM.jpg]

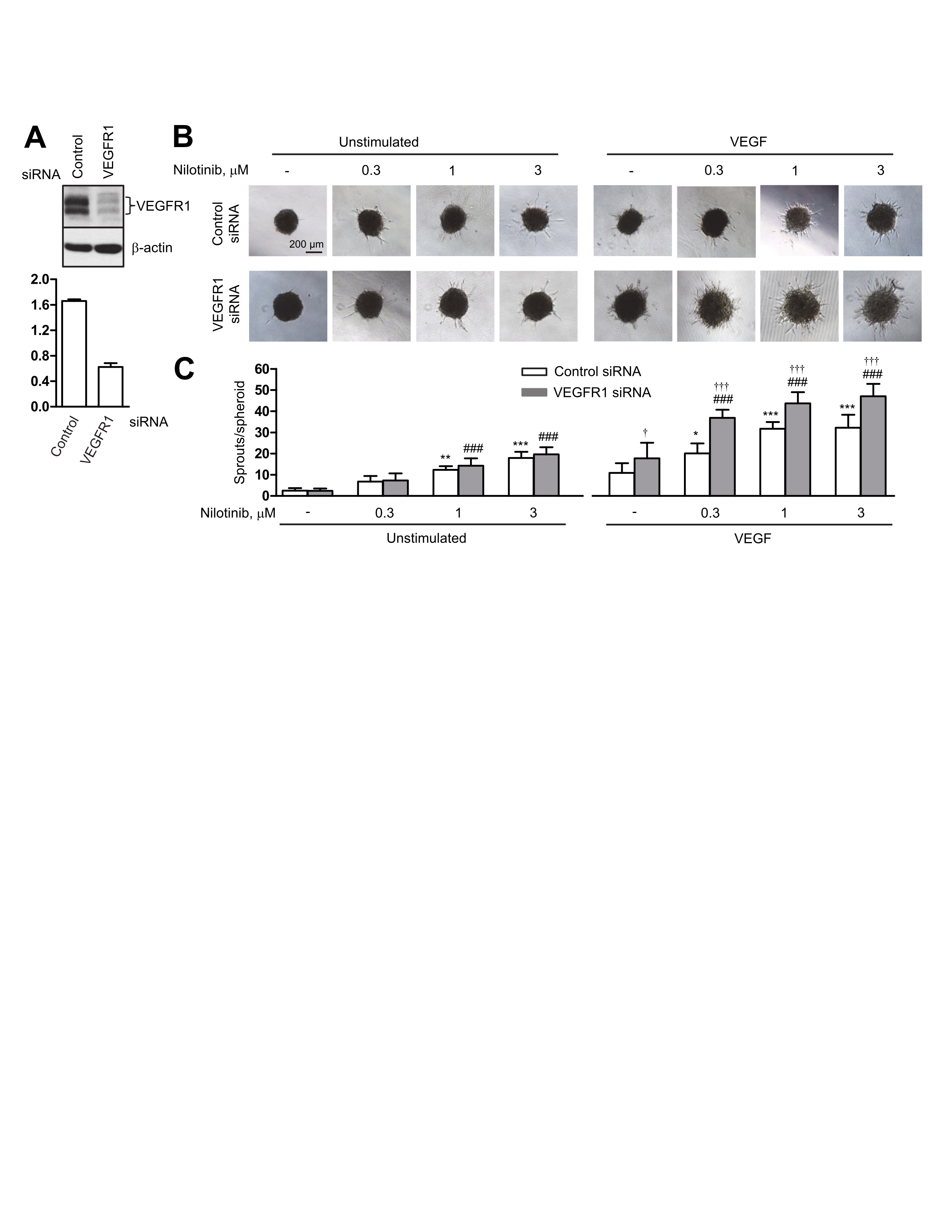

Supplement: Supplementary file 2 — Supplementary file2 (JPG 757 KB)—(A) Human umbilical vein endothelial cells (HUVEC) were transfected with VEGFR1 siRNA for 48 h and harvested for immunoblot analysis to confirm VEGFR1 downregulation. (B, C) HUVEC spheroids, generated from VEGFR1 siRNA-transfected cells, were pre-treated with vehicle (-) or nilotinib at the indicated concentrations for 30 min and then stimulated with VEGF (10 ng/ml, 24 h) or left unstimulated. Representative immunoblot (A) and spheroid images (B) with corresponding analysis (C) are shown. Data are means ± SEM of 3 independent experiments using endothelial cells from different donors. *p<0.05, **p<0.01, ***p<0.001 vs. vehicle (-) treatment in cells transfected with control siRNA; ###p<0.001 vs. vehicle (-) treatment in cells transfected with VEGFR1 siRNA; †p<0.05, †††p<0.001 vs. control siRNA at the corresponding condition [file 11010_2024_5070_MOESM2_ESM.jpg]

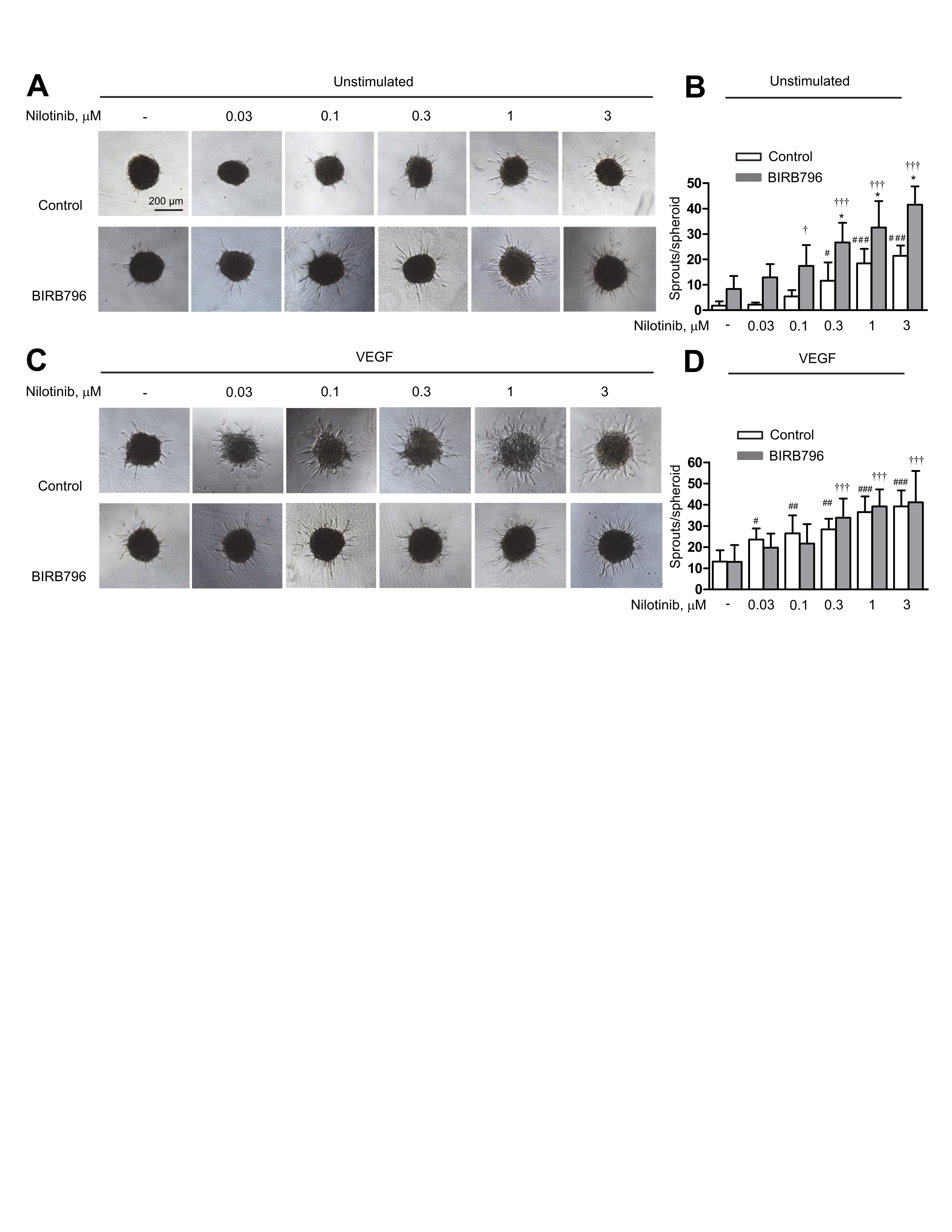

Supplement: Supplementary file 3 — Supplementary file3 (JPG 1224 KB)—Pan-p38 MAPK inhibitor does not affect the pro-angiogenic effect of nilotinib. Human umbilical vein endothelial cells (HUVEC) spheroids were pre-treated with either BIRB796 (10 μM, 30 min) or solvent (control) prior to treatment with vehicle (-) or nilotinib at the indicated concentrations for 30 min. Afterwards, spheroids were left either unstimulated (A–B) or were stimulated with VEGF (10 ng/ml, 24 h) (C–D). Representative images (A, C) and analyses of the number of sprouts per spheroid (B, D) are shown. Data are mean ± SEM of 3 independent experiments using endothelial cells from different donors. *p<0.05 vs. respective control; #p<0.05, ##p<0.01 ###p<0.001 vs. vehicle (-) treatment under control conditions, †††p<0.001 vs. vehicle (-) treatment under BIRB796 treatment conditions [file 11010_2024_5070_MOESM3_ESM.jpg]

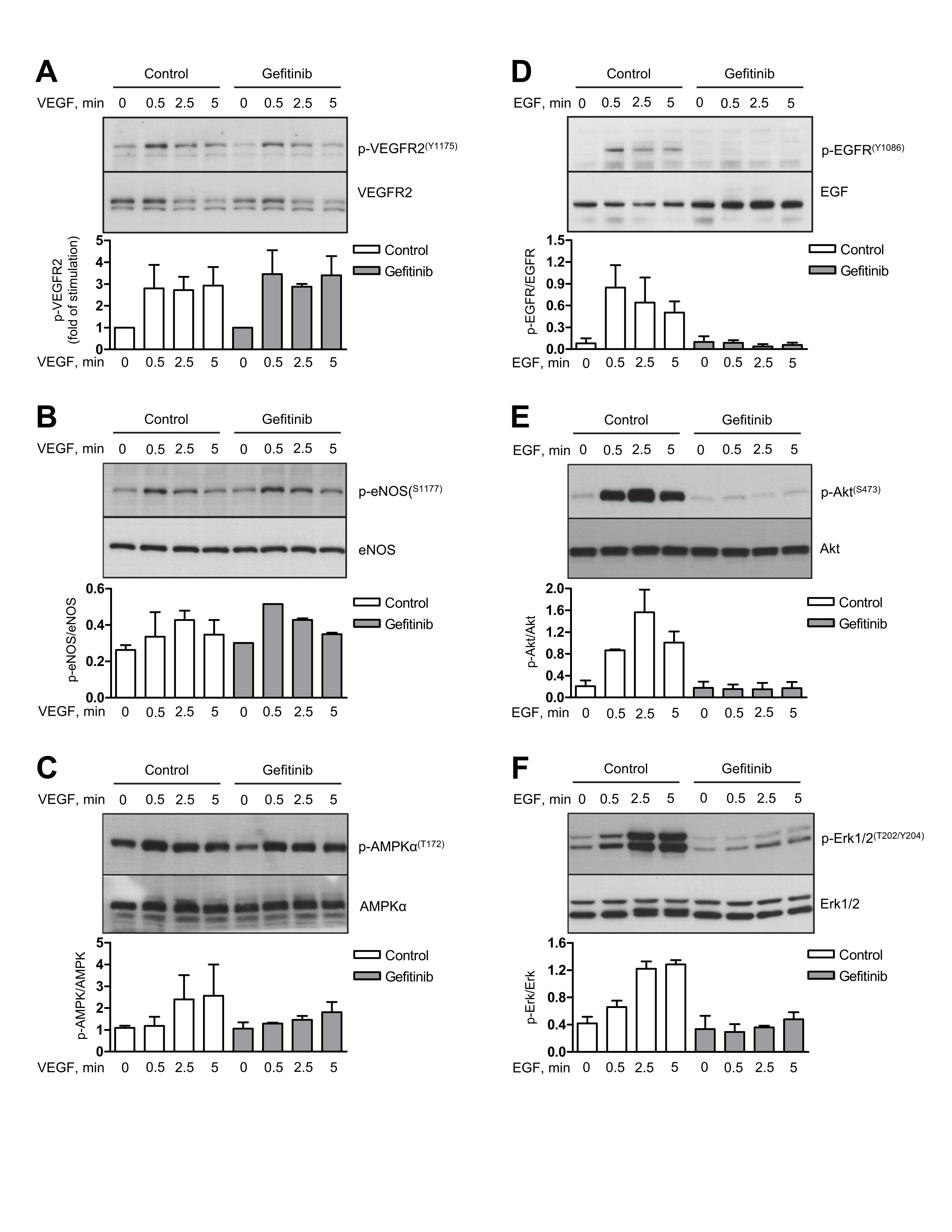

Supplement: Supplementary file 4 — Supplementary file4 (JPG 1077 KB)—Gefitinib inhibits EGFR but not VEGF signalling. Human umbilical vein endothelial cells (HUVEC) were pre-treated with gefitinib (1 μM, 30 min) or vehicle (control) prior to stimulation with either VEGF (50 ng/ml) (A–C) or EGFR (100 ng/ml) (D–F) for the indicated time points; afterwards cells were harvested for immunoblot analysis. Representative images and densitometric analyses are shown. Data are mean ± SEM of 2 independent experiments using endothelial cells from different donors [file 11010_2024_5070_MOESM4_ESM.jpg]
